# The Distribution of Succinoxidase in Various Fractions of the Cytoplasm of Some Normal and Malignant Cells of Mice

**DOI:** 10.1038/bjc.1953.23

**Published:** 1953-06

**Authors:** L. Dmochowski, L. H. Stickland


### THE DISTRIBUTION OF SUCCINOXIDASE IN VARIOUS FRACTIONS OF THE CYTOPLASM OF SOME NORMAL AND MALIGNANT CELLS OF MICE.

L. DMOCHOWSKI AND L. H. STICKLAND.

*From the Department of Experimental Pathology and Cancer Research, School of Medicine, Leeds* 2.

Received for publication March 20, 1953.

THE method, based on mechanical break-up of cells in a suitable medium and followed by separation of the various cell components by means of differential centrifugation, is now extensively used in the study of the distribution of enzymes within cells. In this way the distribution of succinoxidase, among other enzymes, has extensively been studied in some normal and malignant tissues by Hogeboom, Claude and Hotchkiss (1946), Schneider (1946*a*), Hogeboom, Schneider and Pallade (1948), Schneider, Claude and Hogeboom (1948), and Schneider and Hogeboom (1950*a*, 1950*b*).

In connection with these studies experiments were undertaken to ascertain whether any differences in succinoxidase activity exist between normal and malignant mammary tissues of high- and low-breast-cancer-strain mice.

### EXPERIMENTAL.

### *Material.*

Spontaneous mammary tumours of high-breast-cancer strains were taken from mice of Strong A, C3H and RIII strains. Tumours from mice of all three strains were pooled for each experiment. In a single experiment the number of animals used varied from 3 to 16. Necrotic tissue was removed as far as possible from each tumour. RIIIb strain transplantable mammary carcinoma was used as a low-cancer-strain tumour. This tumour originated as a spontaneous carcinoma which developed in a breeding female of RIII strain, deprived of the milk factor by foster nursing by a C57 low-cancer-strain mother. Lactating mammary glands were obtained by sacrificing the mothers of 7- to 14-day-old litters and were taken from RIII and Strong A high-cancer and RIIIb low-cancer-strain mice. For purposes of comparison mouse liver was taken from all strains indiscriminately, and rat liver from Wistar strain rats.

### *Methods.*

The method of fractionation employed started with mechanical rupture of the cells by means of a mechanically driven Potter-Elvehjem glass homogeniser (Potter and Elvehjem, 1936). After a preliminary mincing with scissors, the tissues were homogenised in a volume of 0·25 M sucrose equal to 9 times the weight of tissue. Isotonic sucrose solution was chosen in preference to hypertonic solution, because it was found to prevent agglutination of microsomes (Hogeboom,

Schneider, and Pallade, 1948), shorten the time of fractionation compared with that taken with tissues homogenised in hypertonic sucrose solutions, and at the same time make no appreciable difference to the distribution of enzymes in the different fractions of cells (Schneider and Hogeboom, 1950*a*). In order to deal easily with rather large quantities of tissue, larger glass homogenisers than usual were used, capable of holding 50 ml. of homogenate. They were made by Messrs. R. Cuthbert Ltd., Huddersfield, whose valuable co-operation is gratefully acknowledged. On some occasions as much as 10 g. of tissue was homogenised in a total volume of 50 ml., more sucrose being added later to dilute the homogenate to a final concentration of 10 per cent. This, as was shown by direct comparison, had no effect on subsequent fractionation. It was found in preliminary experiments that maximal liberation of cytoplasmic proteins into the supernatant fluid was achieved by homogenisation lasting for 1 to 2 minutes.

The separation of the cell components was carried out by means of differential centrifugation at a temperature of 2° to 4° C. in a " Spinco " (Specialised Instruments Corporation, Belmont, California) Model E ultracentrifuge, except for the preliminary removal of nuclei, unbroken cells, debris, etc., which was carried out in an ordinary low-speed centrifuge. The centrifugal procedure with some modifications was based on those, originally described by Claude (1943, 1946) and developed by Hogeboom, Claude, and Hotchkiss (1946), Hogeboom, Schneider, and Pallade (1948), Schneider (1946*b*). In some of the later experiments it was based on that of Schneider and Hogeboom (1950*a*). As can be seen in Table I,

TABLE I.—*Centrifugation Procedure.*

Homogenate 2500 g.—2 min.
(3750 r.p.m.)

| | |
|---|---|
| Nuclei—debris (discarded) | " Homogenate," 5000 × g.—10 min. (8000 r.p.m.) |
| Mitochondria, washed (twice) | Supernatant, 15,000 × g.—20 min. (13,410 r.p.m.) |
| Large microsomes, washed (twice) | Supernatant, 130,000 × g.—60 min. (39,460 r.p.m.) |
| Small microsomes, washed (twice) | Final supernatant |

the " submicroscopic " fraction of Schneider and collaborators was subdivided into " large " or faster sedimenting and " small " or slower sedimenting microsomes.

The morphology of each fraction was examined in smears fixed and stained according to Altmann's method with acid anilin fuchsin.

Total protein in each fraction was estimated calorimetrically by the biuret reaction according to the method of Robinson and Hogden (1940). The succinoxidase activity of each fraction was determined by the method described by Umbreit, Burris and Stauffer (1945) and the results expressed as $QO_2$ (N). The total quantity of the enzyme in each fraction was calculated, and expressed as a percentage of that in the homogenate (" homogenate " for the purposes of the present study will be taken as the supernatant remaining after the removal of nuclei and cell debris).

### RESULTS.

The distribution of succinoxidase among the various fractions of normal lactating mammary gland tissue of mice from both high- and low-breast-cancer strains was found to be different from that observed in liver of mice and rats (Table II).

TABLE II.—*Total Succinoxidase Distribution.*

| Fraction. | Type of tissue. | | | |
|---|---|---|---|---|
| | Rat liver. | Mouse liver. | Lactating breast. | |
| | | | High-cancer-strain. | Low-cancer-strain. |
| Homogenate | 100 | 100 | 100 | 100 |
| Mitochondria | 92<br>(73–120)* | 89<br>(67–116) | 57<br>(46–75) | 60 |
| Large microsomes | 1·3<br>(0·8–1·4) | 2<br>(1–5) | 12<br>(7–15) | 8 |
| Small microsomes | 0·3<br>(0·1–0·6) | 0·5<br>(0·2–1·0) | 5<br>(3–8) | 5 |
| Supernatant | 0 | 0 | — | — |
| Yield | 94<br>(75–121) | 92<br>(73–118) | 74<br>(66–90) | 73 |
| Number of experiments | 6 | 4 | 3 | 1 |

* The figures in parentheses indicate the range of values.

The enzyme was found to be concentrated in the mitochondrial fraction of rat and mouse liver, as already reported by Hogeboom, Claude and Hotchkiss (1946), Hogeboom, Schneider and Pallade (1948) and Schneider and Hogeboom (1950*a*) on rat liver and by Schneider and Hogeboom (1950*b*) on mouse liver. A concentration of the enzyme in this fraction was also found in normal lactating breast tissue of high- and low-cancer-strain mice. While the microsomal fractions of rat and mouse liver showed only a small amount of the enzyme, the same fractions of breast tissue revealed an appreciable quantity of it.

In order to establish that this activity was a true property of these fractions, on some occasions the activity was determined after washing and was found to be unaltered.

These results expressed as specific activity of the enzyme in the respective fractions are shown in Table III. As can be seen in Tables II and III, there was no difference in the total amount of activity or in the specific activity of the corresponding fractions of normal lactating mammary gland tissue between high- and low-cancer-strain mice.

TABLE III.—*Specific Activity in Terms of $QO_2$.*

| Fraction. | Type of tissue. | | | |
|---|---|---|---|---|
| | Rat liver. | Mouse liver. | Lactating breast. | |
| | | | High-cancer-strain. | Low-cancer-strain. |
| Homogenate | 453 (230–760)* | 550 (415–770) | 188 (178–200) | 266 |
| Mitochondria | 1708 (725–2350) | 1930 (1490–2480) | 573 (315–920) | 850 |
| Large microsomes | 113 (35–270) | 157 (81–275) | 334 (170–544) | 174 |
| Small microsomes | 23 (6–53) | 37 (12–60) | 118 (74–187) | 157 |
| Supernatant | 0 | 0 | — | — |
| Number of experiments | 6 | 4 | 3 | 1 |

* The figures in parentheses indicate the range of values.

Comparison of the distribution and of the activity of succinoxidase in the various fractions from normal and malignant mammary cells of high- and low-breast-cancer-strains of mice is shown in Tables IV and V. No difference was

TABLE IV.—*Total Succinoxidase Distribution.*

| Fraction. | Type of tissue. | | | |
|---|---|---|---|---|
| | High-cancer-strains. | | Low-cancer-strains. | |
| | Lactating breast. | Breast tumour. | Lactating breast. | Breast tumour. |
| Homogenate | 100 | 100 | 100 | 100 |
| Mitochondria | 57 (46–75)* | 55 (38–71) | 60 | 56 (55–56) |
| Large microsomes | 12 (7–15) | 9 (2–22) | 8 | 8 (5–11) |
| Small microsomes | 5 (3–8) | 3 (1–14) | 5 | 3 (2–3) |
| Supernatant | 0 | 0 | — | 0 |
| Yield | 74 (66–90) | 67 (42–97) | 73 | 67 (63–69) |
| Number of experiments | 3 | 15 | 1 | 2 |

* The figures in parentheses indicate the range of values.

observed between normal and malignant breast tissues in either the high- or the low-cancer-strain mice. Similarly, no difference was found between mammary tumour tissues from the high- and low-cancer-strains examined. As can be seen from Tables IV and V as well as Tables II and III, removal of all particulate components sedimentable at 130,000 times gravity after 90 minutes resulted in complete removal of demonstrable activity in all tissues.

Comparison of results with MSE type and Potter-Elvehjen homogenisers suggested that the activity of microsomes, especially the faster sedimenting, might be due to fragmented mitochondria, which would sediment with the smaller

TABLE V.—*Specific Activity in Terms of $QO_2$.*

| Fraction. | Type of tissue. High-cancer-strains. Lactating breast. | Breast tumour. | Low-cancer-strains. Lactating Breast | Breast tumour. |
|---|---|---|---|---|
| Homogenate | 188 (178–200)* | 148 (78–292) | 266 | 153 (138–167) |
| Mitochondria | 573 (315–920) | 862 (191–1500) | 850 | 582 (520–644) |
| Large microsomes | 334 (170–544) | 291 (39–965) | 174 | 303 (300–305) |
| Small microsomes | 118 (74–187) | 54 (14–113) | 157 | 52 (45–59) |
| Supernatant | 0 | 0 | — | 0 |
| Number of experiments | 3 | 15 | 1 | 2 |

* The figures in parentheses indicate the range of values.

fractions and would be expected to possess the same $QO_2$ as whole mitochondria. This expectation was based on the findings of Hogeboom and Schneider (1950) on the ultrasonic disintegration of rat liver mitochondria. Since the same distribution of enzyme in rat and mouse liver was observed in the present experiments as that reported by other workers, this explanation could be correct only if mitochondria of mouse mammary tissue, both normal and malignant, were more easily broken during homogenisation than those from liver.

Mitochondria were therefore separated from rat liver and from normal and malignant mammary tissue of mice and suspended in 0·25 M sucrose solution at a concentration of the order of 5 mg. protein per 1 ml. These suspensions of isolated mitochondria were then treated in the Potter-Elvehjen homogeniser for 5 minutes under identical conditions, and fractionated into mitochondrial, large, and small microsomal fractions. Determination of the specific activity of the respective fractions in terms of their $QO_2$ values showed that fragments of mitochondria were not devoid of enzyme action (Table VI). The $QO_2$ values of the large and small microsomal fractions recovered from the fragmented mitochondria from all three tissues were found to be of the same order of magnitude as those of the original mitochondria from these tissues. Protein estimations demonstrated that there was no great difference in the ease with which mitochondria from the different tissues could be fragmented under identical conditions (Table VII); if anything, those from liver were more easily broken up.

It therefore appears that the succinoxidase activity found in the large microsome fraction is a property of the microsomes themselves, and this is probably also true of the small microsomes.

TABLE VI.—*Specific Activity of Fragmented Mitochondria in Terms of $QO_2$.*

| | Fraction. | Type of tissue. Rat liver. | Lactating breast. | Breast tumour. |
|---|---|---|---|---|
| | Original mitochondria | 1340 (725–1700)* | 355 | 664 (590–790) |
| Fractions recovered after fragmentation of mitochondria | Mitochondria | 2120 (610–3110) | 420 | 379 (215–462) |
| | Large microsomes | 1030 (560–1330) | 290 | 937 (446–1300) |
| | Small microsomes | 470 (420–550) | 770 | 1977 (220–3770) |
| | Supernatant | 115 (72–160) | — | — |
| | Number of experiments | 3 | 1 | 3 |

* The figures in parentheses indicate the range of values.

TABLE VII.—*Protein Distribution after Fragmentation of Mitochondria.*

| Fraction. | Type of tissue. Rat liver. | Lactating breast. | Breast tumour. |
|---|---|---|---|
| Original mitochondria | 100 | 100 | 100 |
| Mitochondria | 45 (28–56)* | 61 | 58 (22–92) |
| Large microsomes | 12 (5–25) | 19 | 9 (8–10) |
| Small microsomes | 6 (4–11) | 5 | 5 (3–6) |
| Supernatant | 22 (15–35) | 2 | 13 (11–15) |
| Yield | 85 (74–99) | 87 | 85 (49–120) |
| Number of experiments | 3 | 1 | 3 |

* The figures in parentheses indicate the range of values.

In the examination of smears from all fractions a variable number of mitochondria was found in the large microsomal fraction. In the majority of cases, however, this number appeared to be considerably smaller than that seen in smears from the mitochondrial fraction. The evidence from smears is therefore not enough alone to decide to what extent the enzyme activity found in the microsome fraction was due to contamination with mitochondria. Only in a very few cases were occasional mitochondria seen in smears from the small microsome fractions.

The distribution of the cytoplasmic proteins among the different fractions from rat and mouse livers, lactating mammary glands and from mammary tumours is shown in Table VIII. The results presented in Table VIII support the view that the large or faster sedimenting microsome fraction of normal and malignant mammary tissues is not to a great extent contaminated with mitochondrial matter, because, if it were, it should make up a bigger proportion of the total protein.

TABLE VIII.—*Protein Distribution.*

| Fraction. | Type of tissue. | | | | | |
|---|---|---|---|---|---|---|
| | Rat liver. | Mouse liver. | Lactating breast. | | Breast tumour. | |
| | | | High cancer. | Low cancer. | High cancer. | Low cancer. |
| Homogenate | 100 | 100 | 100 | 100 | 100 | 100 |
| Mitochondria | 26 (20–31)* | 25 (19–29) | 21 (16–30) | 23 | 10 (6–17) | 15 (14–15) |
| Large microsomes | 7 (4–9) | 9 (4–13) | 8 (5–11) | 10 | 5 (2–12) | 4 (3–5) |
| Small microsomes | 9 (7–10) | 8 (5–10) | 6 (5–8) | 6 | 10 (5–13) | 8 (7–9) |
| Supernatant | 45 (37–52) | 49 (35–60) | 46 (35–56) | 40 | 60 (47–75) | 62 (50–73) |
| Yield | 87 (73–99) | 91 (63–110) | 82 (65–91) | 79 | 85 (68–99) | 89 (79–110) |
| Number of experiments | 6 | 4 | 3 | 1 | 15 | 2 |

* The figures in parentheses indicate the range of values.

Finally, separation of mitochondria at 8000 times gravity for 10 minutes instead of 5000 times gravity did not lead to any difference in the activity of the mitochondria, and resulted in no appreciable diminution of activity of the large microsome fraction.

### DISCUSSION.

The present experiments have confirmed the original observation of other workers that succinoxidase is almost exclusively localised in the mitochondria of rat liver (Hogeboom, Claude and Hotchkiss, 1946; Hogeboom, Schneider and Pallade, 1948; Schneider and Hogeboom, 1950*a*), and in the mitochondria of mouse liver (Schneider and Hogeboom, 1950*b*). While a concentration of this enzyme has also been found in the mitochondria of normal and malignant mammary cells of mice, an appreciable proportion of this enzyme (varying from 5 to 22 per cent.) has also been found in the large microsomes of these cells, and a small but significant amount (varying from 2 to 14 per cent) in the small microsomes from cells of normal lactating mammary tissue and from mammary tumour tissue of mice. In particular experiments the $QO_2$ of the large microsomes was almost as high as that of the mitochondria, and on the average it was about one-half of that of mitochondria. Small succinoxidase activity in other fractions (microsomal) of rat and mouse liver does appear to be the result of contamination with mitochondria. Hogeboom, Claude, and Hotchkiss (1946) found approximately 7 per cent activity in thrice washed microsomal fraction and 52 per cent in similarly treated mitochondrial fraction of rat liver, isolated in 0·85 per cent NaCl. Similar small amounts of succinoxidase activity (1 to 3 per cent) were found by Hogeboom, Schneider and Pallade (1948) and Schneider and Hogeboom (1950*a*) in the microsomal fraction of rat liver isolated in either hypertonic or isotonic sucrose solutions, and by Schneider and Hogeboom (1950*b*) in the same fraction of mouse liver, isolated in 0·25 M sucrose solution. In the present experiments, an average of 2 per cent of enzyme activity was observed in rat liver and 3 per cent in mouse liver after combining the activity of both, the large and small, microsome fractions. In similar fractions of normal and malignant mammary tissue, however, the enzyme activity was 17 per cent and 12 per cent respectively.

The presence of succinoxidase activity in the microsome fractions of mammary cells might be due to (*a*) contamination with mitochondria or (*b*) the breaking up of mitochondria during homogenisation procedure into smaller fragments which would behave centrifugally as microsomes. Such fragments of mitochondria might be expected to have the same high succinoxidase content as the intact mitochondria. Hogeboom and Schneider (1950) observed partial inactivation of succinoxidase after sonic disintegration of mitochondria.

The possibility of contamination with mitochondria could not be excluded by histological examination alone. Mitochondria were invariably observed in smears of microsome fractions, but in considerably smaller numbers than in the mitochondrial fractions : it is, however, impossible to make such comparisons quantitative. The chief arguments against contamination with whole mitochondria are first, that in rat liver no succinoxidase was found in the microsomes (or negligible quantity), and second, that in a number of experiments with mammary tumour tissues the same mitochondria were sedimented in two parallel ways, at 5000 times gravity and at 8000 times gravity, and in the latter case the activity of the microsomes subsequently obtained was only slightly lower than in the former.

The possibility of contamination of microsome fractions by fragments of broken mitochondria cannot easily be disposed of. Clearly such contamination was not observed in liver microsome fractions, obtained in similar manner as those from normal and malignant mammary tissues. Mammary cell mitochondria might conceivably be more easily broken ; a comparison was therefore made to test experimentally the fragility of mitochondria from various sources. The results showed clearly that mitochondria from normal and malignant mammary cells of mice are not more fragile than those from liver, and if anything rather less. It can therefore be deduced that mammary cell mitochondria are not appreciably fragmented during homogenisation, and that consequently the enzymic activity present in the microsome fractions from these cells is likely to be a true property of microsomes present in these fractions. The ready fragmentability of liver-cell mitochondria in the Potter-Elvehjen homogeniser after they have been separated from other cell components is at a first glance not easily reconciled with the fact that no damage appears to occur to these cellular components during the homogenisation of the tissue. This difference is presumably due to the protection of the mitochondria against friction and damage by the presence in the latter case of larger cell fragments.

The enzyme activity of microsomal fractions of normal and malignant breast tissues remained practically unaltered in spite of washings in isotonic sucrose solution, which again points against the possibility of the enzyme being adsorbed on microsomes.

From the experiments of Price, Miller and Miller (1948), Price, Miller, Miller and Weber (1949*a*, 1949*b*) on rat hepatoma and of Schneider and Hogeboom (1950*b*) on mouse hepatoma, it is now known that in both rat and mouse hepatoma the protein content of the mitochondrial and microsomal fractions is much lower than that in similar fractions of normal rat and mouse liver. Further, it has been shown in these studies that in rat and mouse hepatoma the succinoxidase content of the mitochondrial fraction is lower than that in normal rat and mouse liver, the total activity being 5 times lower and the specific activity 2 times lower, in spite of the same distribution of the enzyme in normal and malignant livers. In the present experiments no significant difference was observed between normal and malignant mammary cells, either in the total amount of succinoxidase or in its distribution between the various fractions of these cells. A curious point, so far unexplained, is that although in liver homogenates 100 per cent recovery of succinoxidase could be obtained in the fractions, in mammary tissues the recovery was much lower, amounting on the average to only 70 per cent.

No difference was found between high- and low-cancer-strain mice either in the protein and succinoxidase content or in the distribution of these in the various fractions, but the mammary tumour tissue in each case showed a reduced content of mitochondria, compared with the normal mammary tissue, which agrees with that found by Schneider and Hogeboom (1950*b*) in mouse hepatomas.

The reliability of the simplified centrifugal procedure used in the majority of the experiments has been tested by the use on three occasions of the more complicated procedure of Schneider and Hogeboom (1950*a*), which gave substantially the same results. It may perhaps be of interest to mention that Stern (1939) demonstrated the association of succinoxidase with particles of microsome size in beef heart muscle.

A small point worthy of mention is that for greater convenience of making the enzyme assays, the samples of washed particles from rat and mouse liver and mammary tissues were kept overnight at —79° C. in the $CO_2$ box. It was found by direct test that this treatment had only a slight effect on their succinoxidase content, and storage at this temperature for 8 days caused a loss of only 20 to 30 per cent of activity, while that of small microsomes remained unaffected. Novikoff and Potter (1948) found that rat liver frozen in liquid air and stored at —20° C., when thawed showed succinoxidase activity reduced by 50 per cent. The difference between these findings and our own may possibly be due to the temperature of storage.

In addition succinoxidase activity was estimated in the various fractions obtained from desiccated mammary tumour tissue, and from fresh tumour tissues after filtration through Berkefeld N candles. Desiccation reduced the $QO_2$ of the homogenate and all the fractions derived from it to one-tenth or less compared with that in corresponding fractions of fresh tissues. Filtration of the homogenate from fresh tissue reduced the protein content of mitochondrial fractions and of the large microsome fraction greatly, without affecting their $QO_2$.

### SUMMARY.

(1) Succinoxidase in mammary cells of mice shows a different distribution among the particulate fractions (mitochondria, large microsomes, and small microsomes) from that found in liver cells of rats and mice. The large microsomes contain an appreciable amount of the enzyme.

(2) No difference in succinoxidase content and its distribution in the various fractions could be detected between normal lactating mammary tissue and mammary tumours of mice, whether from high- or low-cancer-strains.

(3) Further, no difference in this respect could be detected in mammary cells whether normal or malignant between high- and low-cancer-strains of mice.

### REFERENCES.

CLAUDE, A.—(1943) *Science*, **97**, 451, 1943.—(1946) *J. exp. Med.*, **84**, 51.
HOGEBOOM, G. H., CLAUDE, A., AND HOTCHKISS, R. D.—(1946) *J. Biol. Chem.*, **165**, 615.
*Idem*, AND SCHNEIDER, W. C.—(1950) *Nature*, **166**, 302.
*Iidem* AND PALLADE, G. E.—(1948) *J. Biol. Chem.*, **172**, 619.
NOVIKOFF, A. B., AND VAN POTTER, R.—(1948) *Ibid.* **173**, 223.
POTTER, V. R., AND ELVEHJEM, C. A.—(1936) *Ibid.*, **114**, 495.
PRICE, J. M., MILLER, E. C., AND MILLER, J. A.—(1948) *Ibid.*, **173**, 345.
*Iidem* AND WEBER, G. M.—(1949*a*), *Cancer Res.*, **9**, 96.—(1949*b*) *Ibid.*, **9**, 398.
ROBINSON, H. W., AND HOGDEN, C. G.—(1940) *J. Biol. Chem.*, **135**, 727.
SCHNEIDER, W. C.—(1946*a*) *Cancer Res.*, **6**, 685.—(1946*b*) *J. Biol. Chem.*, **165**, 585.
*Idem*, CLAUDE, A., AND HOGEBOOM, G. H.—(1948) *Ibid.*, **172**, 451.
*Idem*, AND HOGEBOOM, G. H.—(1950*a*) *Ibid.*, **183**, 123.—(1950*b*) *J. nat. Cancer Inst.*, **10** 969.
STERN, K. G.—(1939) *Cold Spring Harbor Symp. Quant. Biol.*, **7**, 312.
UMBREIT, W. W., BURRIS, R. H., AND STAUFFER, J. F.—(1945) 'Manometric Techniques and Related Methods for Study of Tissue Metabolism'. Minneapolis. (Burgess.)


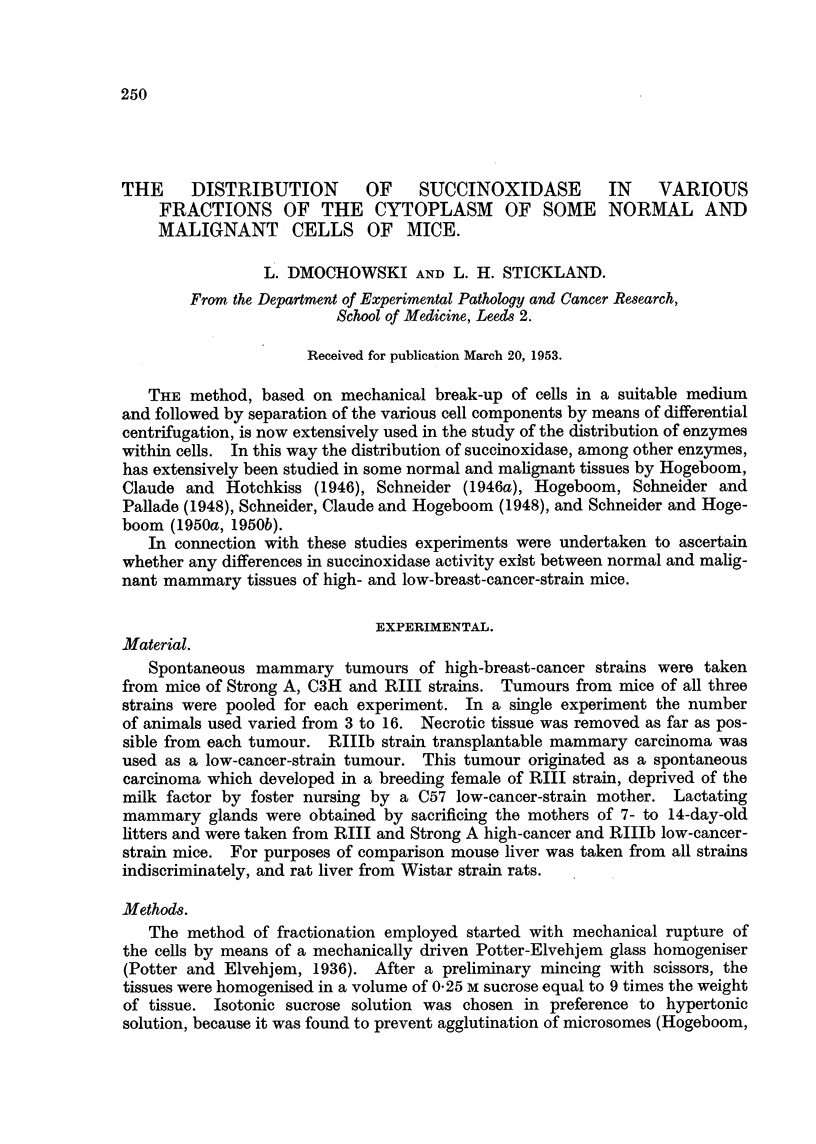

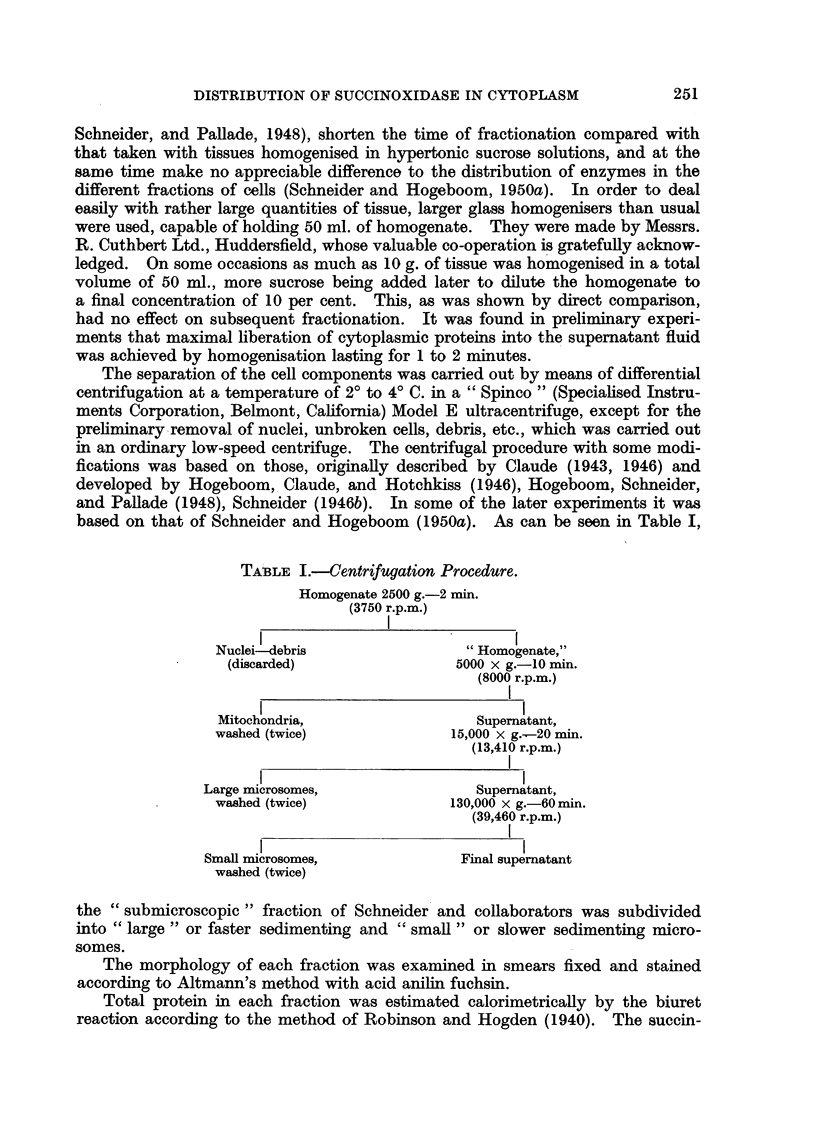

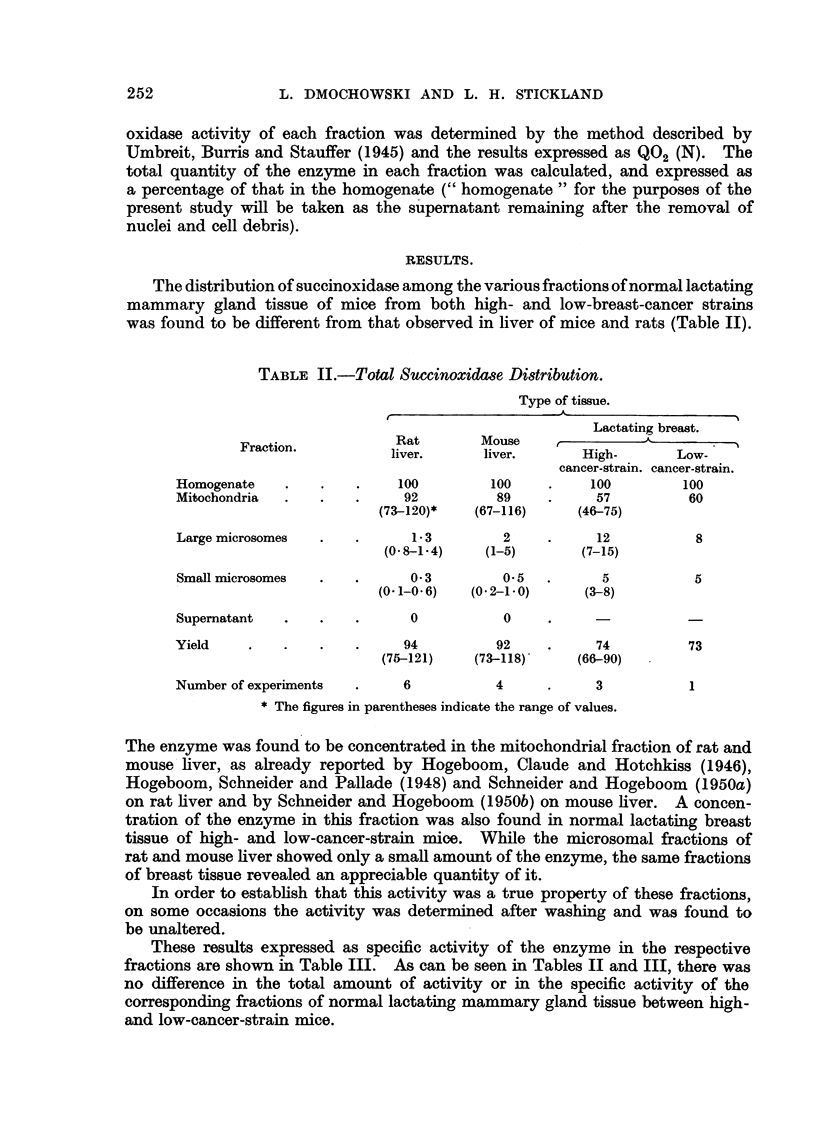

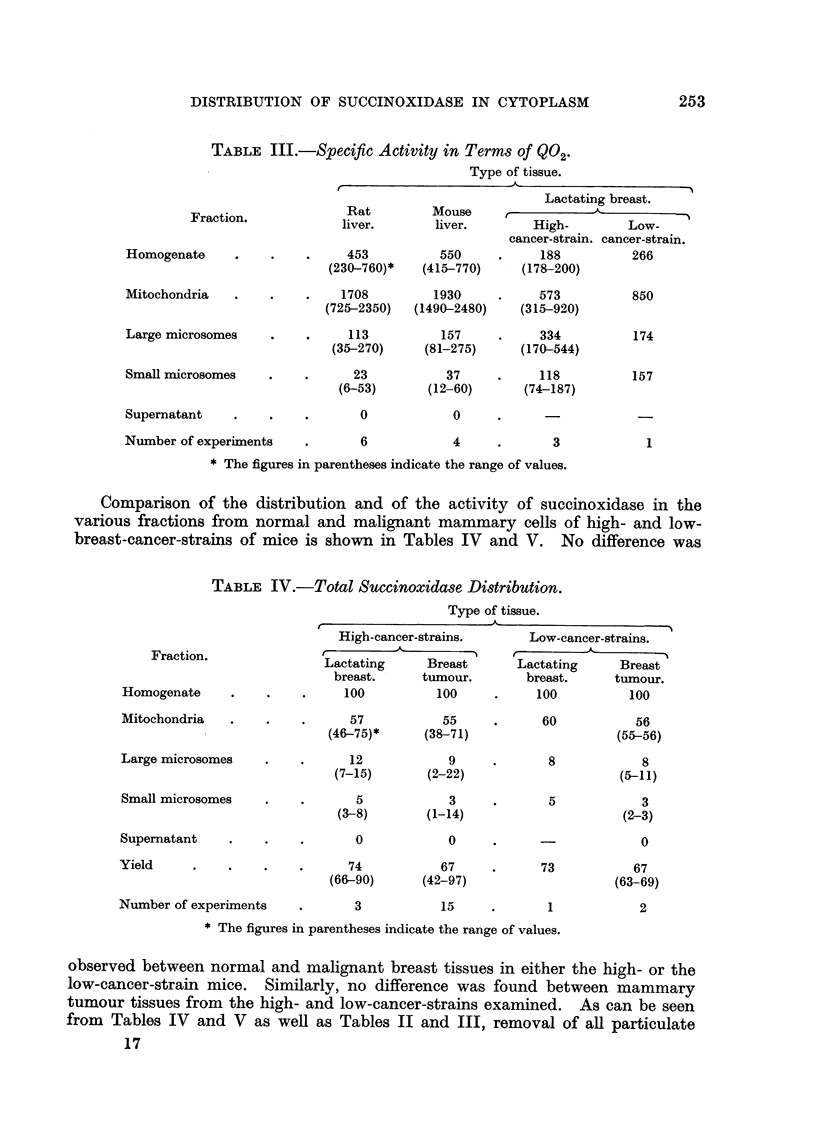

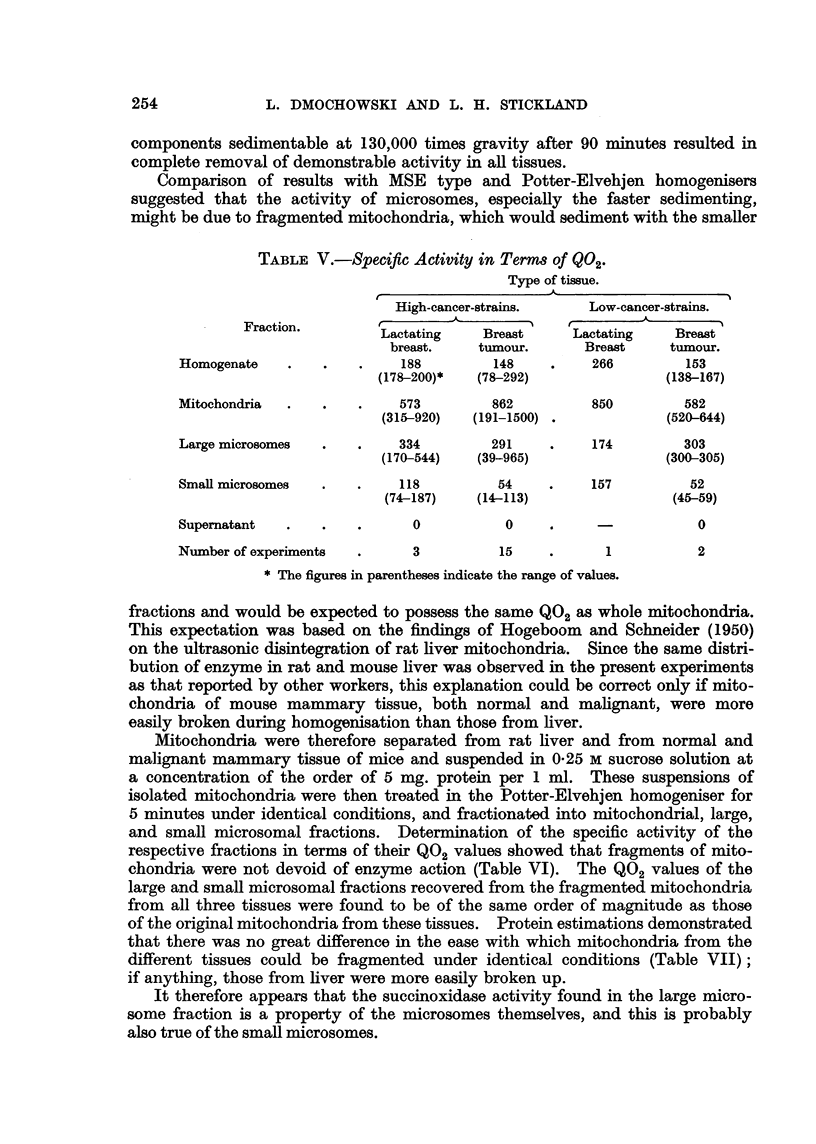

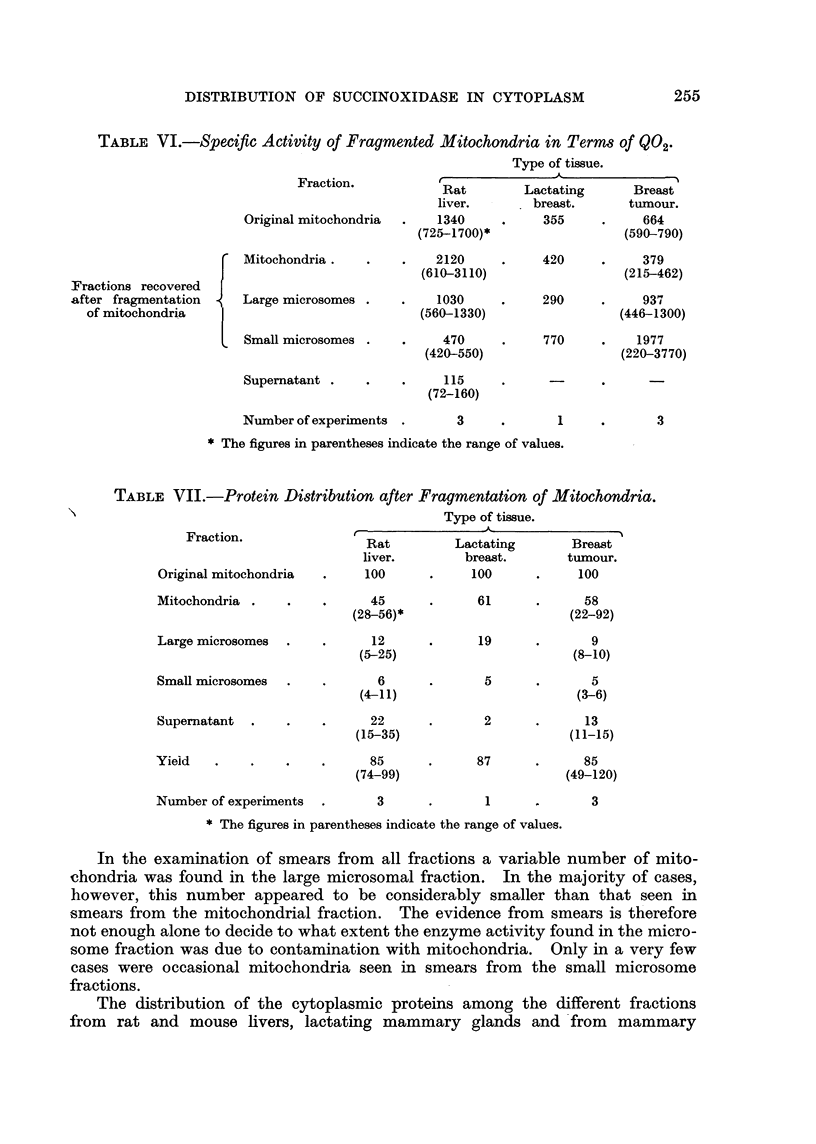

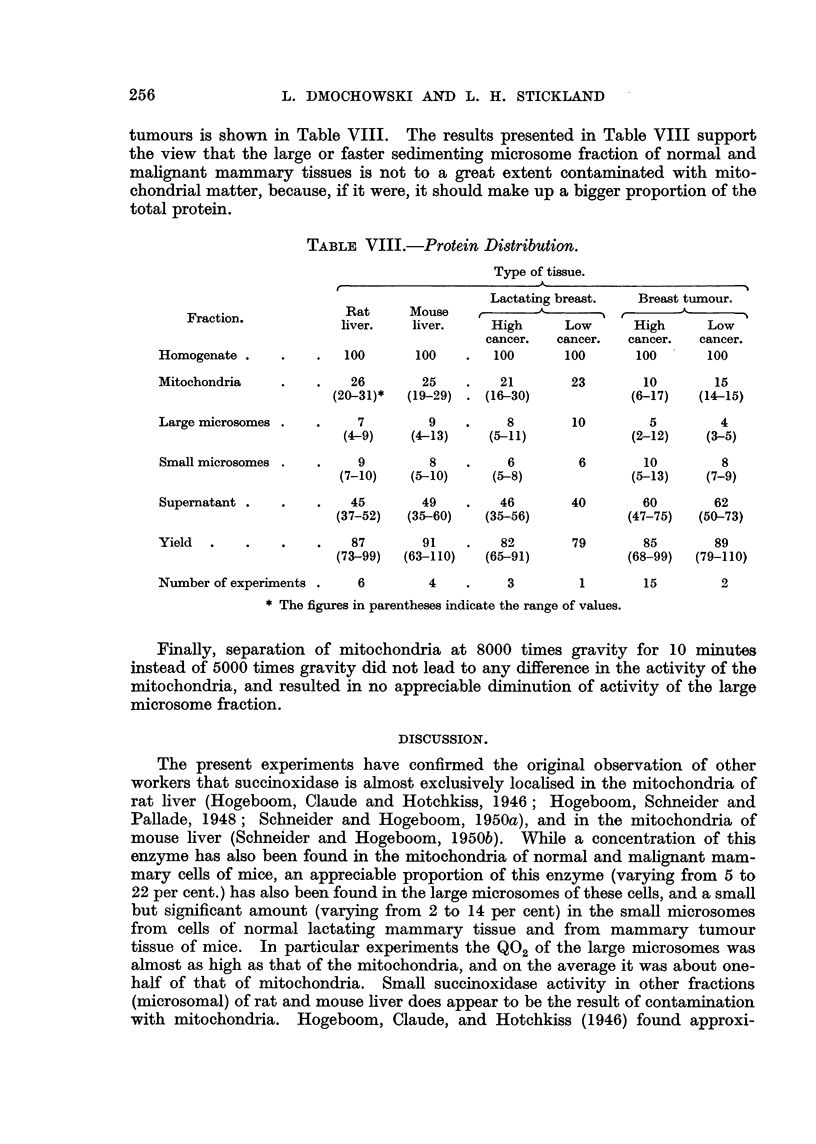

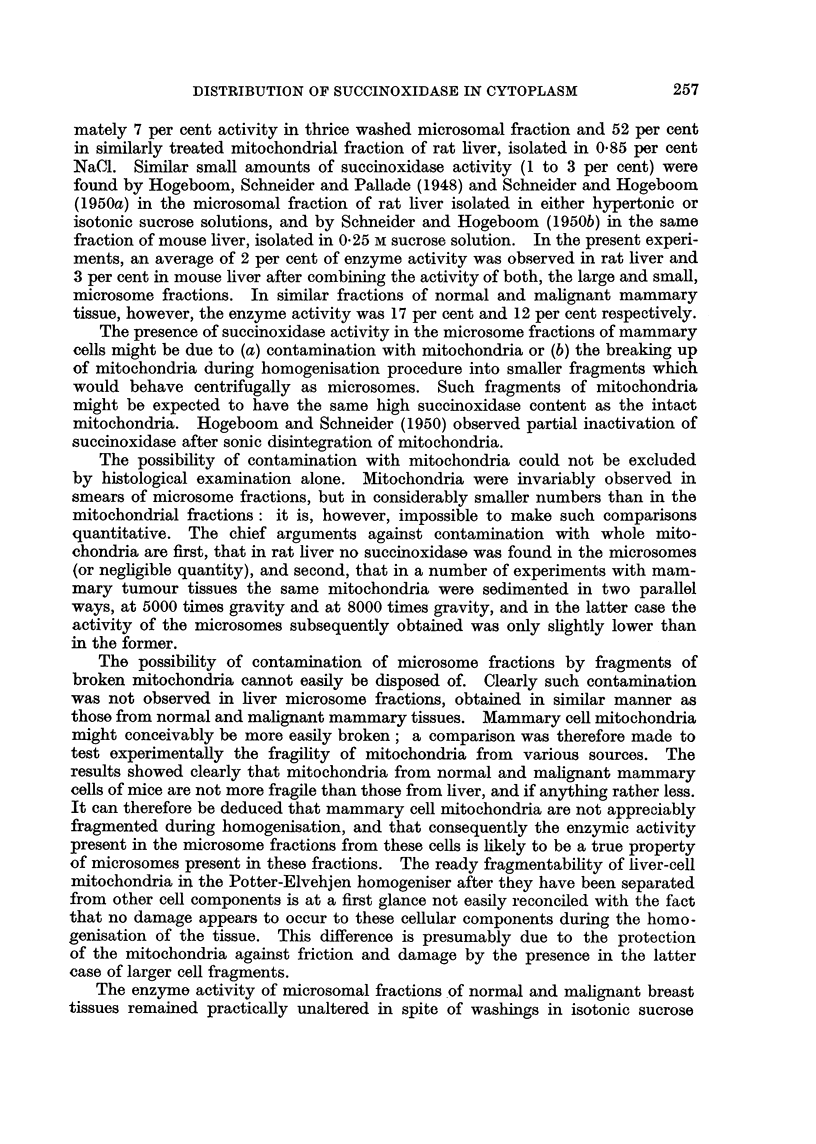

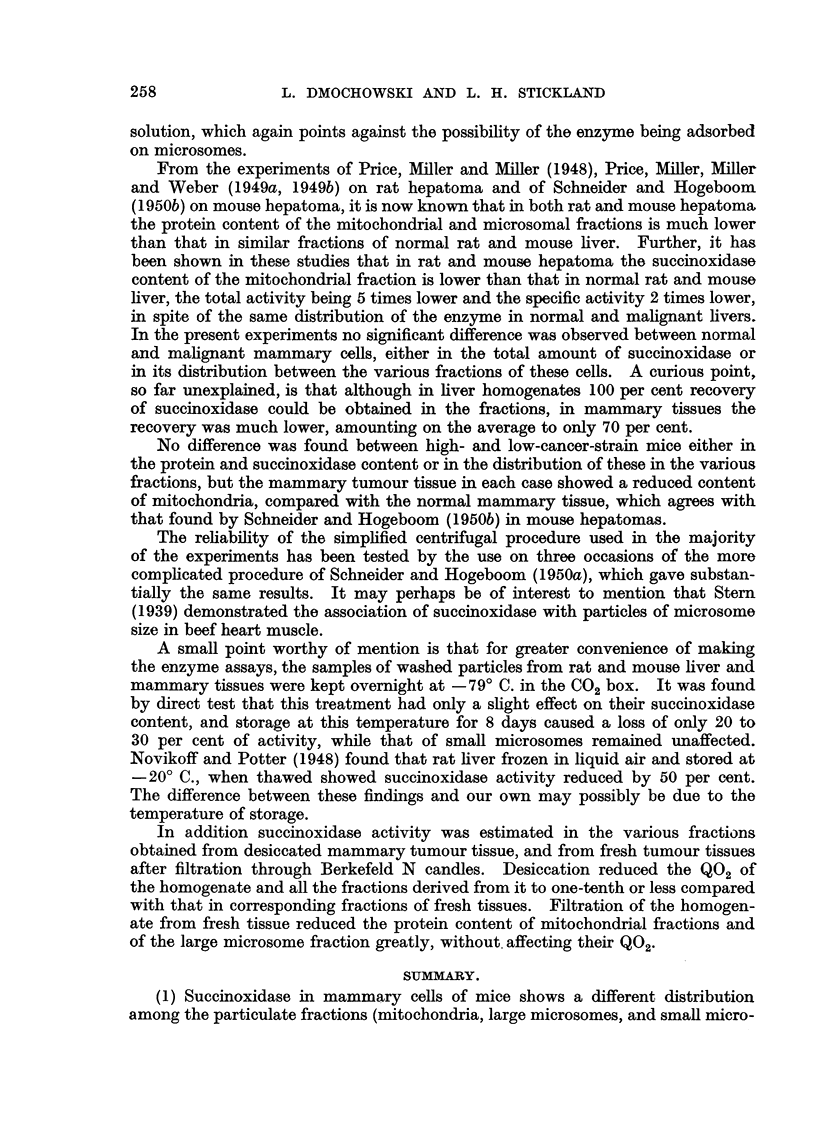

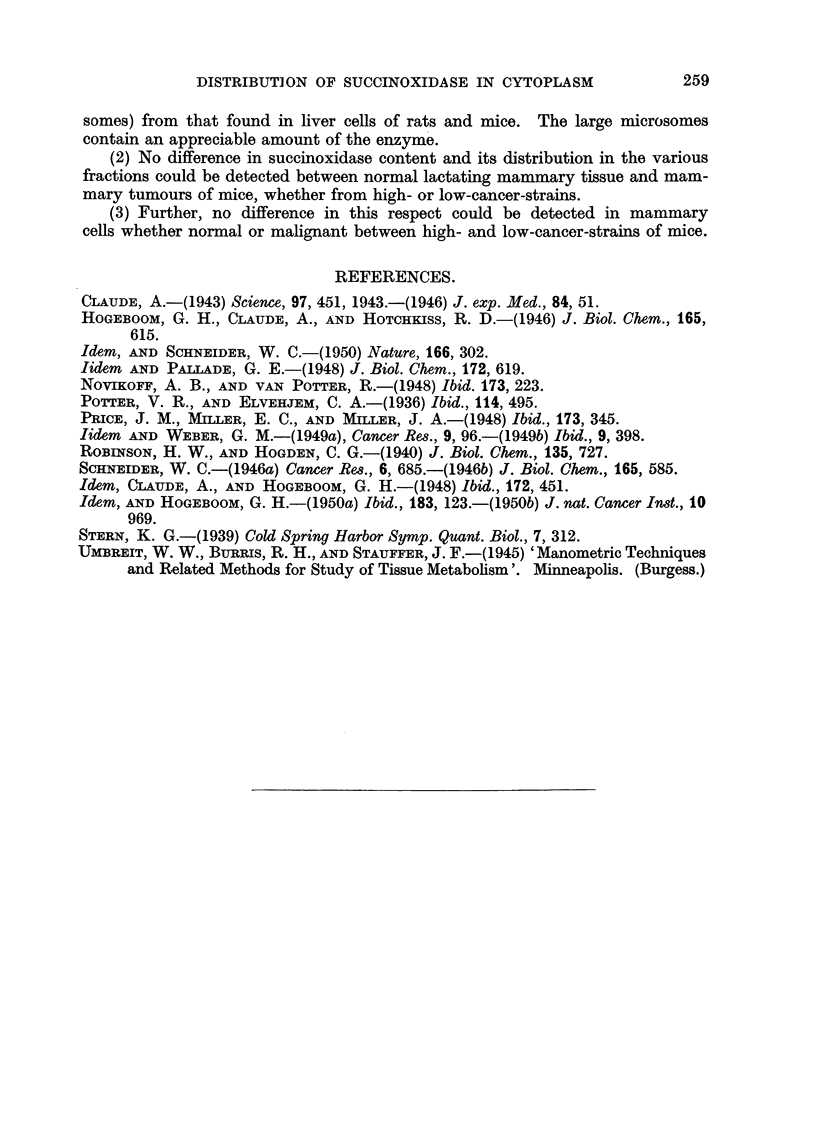


## References

[OCR_00648] Claude A. (1943). THE CONSTITUTION OF PROTOPLASM.. Science.

